# Reunion of Parts Severed from the Body

**Published:** 1893-05

**Authors:** 


### Reunion of Parts Severed from the Body.

At a meeting of the Clinical Society of Maryland, Dr. J. T. M. Finney related a case of severed fingers reapplied seven hours after the accident with perfect union, and recovery of motion and sensation. On January 2, 1890, the patient, a machinist by trade, came to the John Hopkins Hospital about half past 12 o'clock, giving the following history: He was a machinist by trade and was running the engine in the absence of the regular engineer in a tin shop. He went to work about five o'clock that morning, and a little later, while going about a machine used for chopping blocks of tin, he dropped something, and while stooping down to pick it up his hand slipped under the knife, and the ends of the middle and ring fingers were cut off. The middle finger was cut off just beyond the last joint. The joint was opened. The ring finger was cut off just above the root of the nail. This occurred, the man said, about half past five o'clock. He wrapped up the stumps and went home, where his wife covered the wounds with beeswax. He arrived at the hospital at the time previously stated. I asked him where the stumps of the fingers were and he produced them, wrapped up in a piece of newspaper. They were very cold, almost frozen. I placed them in a basin of warm water, using no antiseptic, because bichloride or carbolic acid might cause a layer of coagulation necrosis and prevent union. I scrubbed up the stumps of the fingers with a 1-2000 warm bichloride solution, then I carefully rinsed them off in warm water. This process consumed at least half an hour. Then I took a shaving off the ends of the fingers, so as to have a perfectly fresh surface. The stumps were treated in the same manner. The bone was scraped. I sewed them on, using four stitches in each case. I then applied strips of crepe lisse with collodion the whole length of the fingers on each side. These held the severed portions in exact apposition. Then I used other strips around the fingers, binding them together, and then applied a palmar splint and used a large absorbent dressing. He came back in a week, and when the dressing was removed the fingers looked very well. I reapplied the dressing and told him to report in another week. Dr. Brockway saw the case on his return at the end of the second week. He took out the stitches and removed the dressing, and said there was no doubt but that the fingers had united, and that the man seemed to have sensation at the ends of his fingers, although he thought that this sensation might have been transmitted. The man then disappeared entirely from view. He returned about a month ago with an injury to his other hand. It is difficult to say, at first sight, which hand was injured. There was a slight motion in the joint which was opened, and the sensation in the fingers is perfect.

Dr. Randolph Winslow added: This case of Dr. Finney's calls to my mind a case which I had about fifteen years ago. I was called one day to see a woman who followed the occupation of an upholstress. She had chopped the end of her thumb off with a hatchet, perhaps half an hour before I saw her. Upon inquiry about the missing piece, I was told that it was about the floor somewhere. I hunted it up, cleaned it, put it on with adhesive strips, and it is there to this day.

It is rather an important matter that we should replace these lost parts, and in many cases we will have success. I have a number of times replaced parts which were essentially cut off, attached by a minute portion of the skin, with successful union. —*Maryland Med. Jour.*

